# Prospective Neuropsychological and Plasma Biomarker Changes in Treatment-Naïve People Living with HIV After Antiretroviral Treatment Initiation

**DOI:** 10.3390/biomedicines13071704

**Published:** 2025-07-12

**Authors:** Charalampos D. Moschopoulos, Evangelia Stanitsa, Konstantinos Protopapas, Akrivi Vatsi, Irene Galani, Henrik Zetterberg, Ion Beratis, Paraskevi C. Fragkou, Sotirios Tsiodras, Dimitra Kavatha, Antonios Papadopoulos, Sokratis G. Papageorgiou, Anastasia Antoniadou

**Affiliations:** 1 Fourth Department of Internal Medicine, School of Medicine, Attikon University Hospital, National and Kapodistrian University of Athens, 12462 Athens, Greece; kprotopapas@hotmail.com (K.P.); egalani@med.uoa.gr (I.G.); tsiodras@med.uoa.gr (S.T.); dimitra.kavatha@gmail.com (D.K.); antipapa@med.uoa.gr (A.P.); ananto@med.uoa.gr (A.A.); 2 First Department of Neurology, Aiginition University Hospital, National and Kapodistrian University of Athens, 11528 Athens, Greece or eva.st.92@gmail.com (E.S.); ionas96@hotmail.com (I.B.); sokpapa@med.uoa.gr (S.G.P.); 3 Department of Neurosurgery, Evangelismos Hospital, National and Kapodistrian University of Athens, 10676 Athens, Greece; avatsi@med.uoa.gr; 4 Department of Psychiatry and Neurochemistry, Institute of Neuroscience and Physiology, The Sahlgrenska Academy at the University of Gothenburg, 405 30 Gothenburg, Sweden; henrik.zetterberg@clinchem.gu.se; 5 Clinical Neurochemistry Laboratory, Sahlgrenska University Hospital, 431 80 Mölndal, Sweden; 6 Department of Neurodegenerative Disease, UCL Institute of Neurology, University College London, London WC1N 3BG, UK; 7 UK Dementia Research Institute at UCL, University College London, London WC1N 3AR, UK; 8 Hong Kong Center for Neurodegenerative Diseases, Hong Kong, China; 9 Wisconsin Alzheimer’s Disease Research Center, University of Wisconsin School of Medicine and Public Health, University of Wisconsin-Madison, Madison, WI 53726, USA; 10 Centre for Brain Research, Indian Institute of Science, Bangalore 560012, India; 11 Psychology Department, American College of Greece, Deree, 6 Gravias Street, 15342 Athens, Greece; 12 Internal Medicine Department, Aegli Medical Clinic, 19003 Markopoulo, Greece; evita.fragou@gmail.com

**Keywords:** neuroHIV, HIV, biomarkers, monocyte activation, cART, HIV-associated neurocognitive disorder

## Abstract

**Introduction**: Human immunodeficiency virus (HIV)-associated neurocognitive impairment (NCI) remains a concern despite combination antiretroviral therapy (cART), with cognitive problems often persisting even after viral suppression. The mechanisms underlying neurocognitive deterioration in people living with HIV (PLWH) and the role of plasma biomarkers remain unclear. This study aims to evaluate neurocognitive trajectories and biomarker changes in a real-world cohort of newly diagnosed PLWH initiating cART in Greece. **Methods**: This prospective, single-center study assessed neuropsychological performance and plasma biomarkers in treatment-naïve PLWH at baseline and 18 months after cART initiation. HIV-associated neurocognitive disorder (HAND) was classified using the Frascati criteria, and plasma biomarkers of inflammation and monocyte activation were measured. Correlations between biomarkers and cognitive performance were analyzed. **Results**: A total of 39 treatment-naïve PLWH were enrolled in this study. At baseline, 45.7% of participants met criteria for HAND, predominantly, asymptomatic neurocognitive impairment (ANI). Over 18 months, neurocognitive function improved, particularly in speed of information processing, executive function, and visuospatial ability, while verbal fluency, fine motor dexterity, and attention/working memory remained unchanged. Biomarkers of inflammation and monocyte activation decreased following cART, except for neopterin, which increased (10.6 vs. 13 ng/mL, *p* = 0.002), and plasma NFL (7.5 vs. 7.2 pg/mL, *p* = 0.54), which remained stable. A negative correlation between monocyte activation markers and cognitive performance was observed only at follow-up, suggesting that systemic inflammation may mask these associations in untreated PLWH. **Conclusions**: Early cART initiation supports neurocognitive recovery and reduces immune activation in PLWH. The observed correlation between cognitive performance and monocyte activation markers after viral suppression highlights the potential utility of plasma biomarkers in predicting cognitive impairment.

## 1. Introduction

Human immunodeficiency virus (HIV) enters the brain early in the course of infection and establishes a latent reservoir [1]. HIV persistence in the central nervous system (CNS) leads to long-term effects on neuropsychological health, which may present even in people who achieve viral suppression after combined antiretroviral treatment (cART) initiation [2]. Chronic immune activation, HIV compartmentalization in the CNS, microbial translocation, blood–brain barrier (BBB) dysfunction, cART neurotoxicity, CNS viral escape, and legacy effects of uncontrolled HIV replication in the brain are only a few of the proposed pathophysiological mechanisms contributing to neurocognitive issues in people living with HIV (PLWH) [3].

The current classification for HIV-associated neurocognitive diseases (HAND) is based on the Frascati criteria, which include a preclinical stage (asymptomatic neurocognitive impairment—ANI), a stage of intermediate severity (mild neurocognitive disease -MND), and an advanced stage (HIV-associated dementia—HAD) [4]. This classification is based on the performance on neuropsychological testing, the presence and severity of symptoms, and the exclusion of other conditions that could account for neurocognitive impairment (NCI). Based on these criteria, HAND remains prevalent in the cART era, as it affects about 20–50% of PLWH [5,6]. Besides neuropsychological assessment, researchers have long endeavored to ground HAND diagnosis on biological indices, which may also provide evidence regarding HAND mechanisms, progression, and prognosis. To this end, increased inflammation and neuronal damage biomarkers in cerebrospinal fluid (CSF) and plasma have been investigated and found to be associated with HAND, especially with its more severe forms [7,8].

cART has revolutionized the management and prognosis of HIV infection, and PLWH are now expected to have a near normal life expectancy [9]. Consequently, conditions associated with ageing, including neurocognitive impairment, are expected to significantly affect this population. Multimodal neurocognitive assessment is increasingly being considered in cognitive impairment investigation in PLWH, especially in the face of multimorbidity and differential diagnosis challenges [10,11]. Moreover, the effect of viral suppression and immune reconstitution that follow cART initiation on the brain has not yet been fully explored.

In this prospective, pre-post study, we investigated neuropsychological and plasma biomarker changes in a real-world cohort of recently diagnosed and treatment-naïve PLWH initiating cART over an 18-month period.

## 2. Materials and Methods

### 2.1. Study Design

This is a prospective, observational, single-center study with an 18-month follow-up period. We prospectively enrolled participants with recent HIV infection diagnosis, from July 2019 to February 2022. Study enrollment was paused for 6 months during the first phase of the COVID-19 pandemic, as neuropsychological assessment and neuroimaging were not feasible. We enrolled treatment-naïve PLWH upon diagnosis and before cART initiation. Study procedures included a comprehensive neuropsychological assessment and peripheral biomarker measurement. Participants should have completed all assessments within 3 months after treatment initiation. Flexibility was considered important to allow participants to recover from the stress resulting from the recent HIV diagnosis that could confound the neuropsychological assessment. The same procedures were repeated after an 18-month follow-up period. All study assessments took place in the HIV Outpatient Clinic of University General Hospital ‘Attikon’, Athens, Greece. The study was approved by the Institutional Ethics Committee of University General Hospital ‘Attikon’ (ID: 572/23-8-2018 and 528/29-9-2020).

### 2.2. Participants

Newly diagnosed PLWH were consecutively enrolled in the study if they were >18 years old, had not previously received cART (pre-exposure prophylaxis was not available in Greece during the enrollment period), and had Greek as their native language. Exclusion criteria were (a) previous diagnosis of major psychiatric illness (not including well-treated depression and/or anxiety), (b) CNS infection or space-occupying disease, (c) neurodegenerative or cerebrovascular disease, (d) alcohol use disorder, (e) substance use disorder, (f) hepatitis C virus (HCV) infection, and (g) pregnancy. All study participants were informed in detail about the study procedures and gave written informed consent. Anonymized participant data were collected and analyzed, and the research was conducted in accordance with the World Medical Association Declaration of Helsinki. This study is reported in accordance with the STROBE guidelines for cohort studies.

### 2.3. Study Procedures

#### 2.3.1. Demographics and Routine Laboratory Exams

At baseline, a comprehensive history was taken by each participant, including age, gender, HIV infection characteristics (estimated time and mode of transmission), comorbidities (cardiovascular disease, diabetes mellitus, and hypertension), previous or current sexually transmitted infections, habits (smoking, alcohol consumption, and psychoactive drug use), neuropsychiatric conditions, and years of typical education. Medical records were searched to retrieve information about the CDC stage of infection, CD4+ and CD8+ T cell count, HIV viral load, and antiretroviral regimen. Routine laboratory exams also included lipid panel, syphilis serology, vitamin B12, and TSH measurements. Demographics and laboratory exams were also assessed during the follow-up visit.

#### 2.3.2. Neuropsychological Assessment

An extensive neuropsychological battery was used for the assessment of cognitive functions. The selected neuropsychological tests (Table 1) aimed to evaluate seven cognitive domains as suggested by the Frascati criteria [4]. The Frascati criteria classify HAND into asymptomatic neurocognitive impairment (ANI; ≥2 cognitive domains with z-scores ≤ −1 but no functional impairment), mild neurocognitive disorder (MND; ≥2 domains with Z-scores ≤ −1 and mild functional impairment), and HIV-associated dementia (HAD; ≥2 domains with Z-scores ≤ −2 and marked functional impairment). The criteria also propose using clinical ratings to account for multiple ability domains tested and multiple test measures within each domain in order to assign a HAND diagnosis [12]. To this effect, raw scores were transformed into z-scores, based on the best available normative data, and then into T-scores to facilitate qualitative analysis using a 9-point clinical rating scale for neuropsychological measures. A clinical rating of 5 serves as the demarcation point for cognitive decline. Scores decreasing from 4 (borderline) to 1 indicate better cognitive functioning, while scores increasing from 6 to 9 signify rising levels of cognitive impairment. For instance, a score of 6 denotes mild-to-moderate impairment, whereas a score of 9 reflects severe impairment. The Global Deficit Score (GDS) was also used to quantitively summarize the results of neuropsychological testing [13]. The GDS ranges from 0 to 5, with higher scores reflecting greater levels of neurocognitive impairment, with a cutoff of ≥0.5 signifying low cognitive performance. Normative data from a Greek population were used for the following tests: Montreal Cognitive Assessment (MoCA), Greek Verbal Learning Test (GVLT), Trail Making Test Part A and B, Stroop test, and semantic and phonemic verbal fluency tasks. For tests with no published normative data in a Greek population, including Brief Visuospatial Memory Test (BVMT), Symbol Digit Modalities Test (SDMT), Judgement of Line Orientation (JLO), Letter Number Sequencing (LNS), and Spatial Span, we used an appropriate control group of healthy Greek individuals, matched for age and years of typical education.

Depression and anxiety were evaluated using the Hospital Anxiety and Depression Scale (HADS) [23]. For the evaluation of functionality of daily living, Instrumental Activities of Daily Living (IADL) [24] and Functional Activities Questionnaire (FAQ) [25] were administered. These assessments were completed by the participants, as informants were not present during study visits.

#### 2.3.3. Plasma Biomarkers

Plasma levels of inflammatory biomarkers, including CD14, CD163, IL-1β, IL-6, IL-10, TNF-α, MCP-1/CCL2, MIP-1β, S100B, and neopterin, were measured using enzyme-linked immunosorbent assays (ELISA). Blood samples were collected in EDTA tubes and centrifuged at 1500× *g* for 10 min at 4 °C, and the plasma was aliquoted and stored at −80 °C until analysis to avoid repeated freeze–thaw cycles. The assays were conducted with DuoSet^®^ ELISA kits (R&D Systems, Minneapolis, MN, USA) according to the manufacturer’s instructions. The specific kits used for each biomarker are as follows: Human CD14 (DY383), Human CD163 (DY1607), Human IL-1β (DY201), Human IL-6 (DY206), Human IL-10 (DY217B), Human TNF-α (DY210), Human CCL2/MCP-1 (DY279), Human CCL4/MIP-1β (DY271), and Human S100B (DY1820). Neopterin levels were measured using the Human Neopterin kit (EH3413, Wuhan Fine Biotech Co., Wuhan, China). The assays were performed in duplicates, and all measurements were carried out within the detection ranges specified by the manufacturers. Plasma NFL concentration was measured using the single molecule array (Simoa) NF-light assay on an HD-X platform (Quanterix, Billerica, MA, USA), as previously described [26]. All samples were measured in one round of experiments using one batch of reagents with baseline and follow-up samples side by side on the same plate to minimize variation. The measurements were performed by board-certified laboratory technicians who were blinded to clinical data. Intra-assay coefficients of variation (estimated using internal quality control samples in the beginning and end of each plate) were below 10%.

### 2.4. Statistical Analysis

Descriptive statistics were used to summarize participant characteristics at baseline and follow-up. Continuous variables were expressed as mean ± standard deviation (SD) or median (interquartile range [IQR]) depending on data distribution, while categorical variables were presented as frequencies and percentages. The Shapiro–Wilk test was used to assess normality. Paired *t*-tests or Wilcoxon signed-rank tests were used to compare neuropsychological test scores and biomarker levels between baseline and follow-up. Between-group comparisons were performed using independent *t*-tests or Mann–Whitney U tests for continuous variables and chi-square or Fisher’s exact tests for categorical variables. Spearman correlation coefficients were calculated to examine associations between cognitive performance and biomarker levels. Effect sizes were calculated as Cohen’s d for parametric tests and as rank-based *r* for non-parametric tests, as appropriate. According to conventional benchmarks, Cohen’s d values of 0.2, 0.5, and 0.8 were interpreted as small, medium, and large effects, respectively. For effect size *r*, values of 0.1, 0.3, and 0.5 were considered indicative of small, medium, and large effects. To account for attrition during follow-up, we conducted a post hoc sensitivity analysis using the Last Observation Carried Forward (LOCF) imputation method. For participants lost to follow-up, baseline neurocognitive scores were carried forward to the follow-up timepoint. This approach assumes no change in cognitive function in those who did not complete follow-up assessments. A two-sided *p*-value < 0.05 was considered statistically significant. Statistical analyses were conducted using SPSS (IBM SPSS Statistics, Chicago, IL, USA, version 23) and correlation heatmaps were created using GraphPad Prism version 10.4.1 (GraphPad Software, LLC, Boston, MA, USA).

## 3. Results

### 3.1. Participant Characteristics

**Figure 1:**
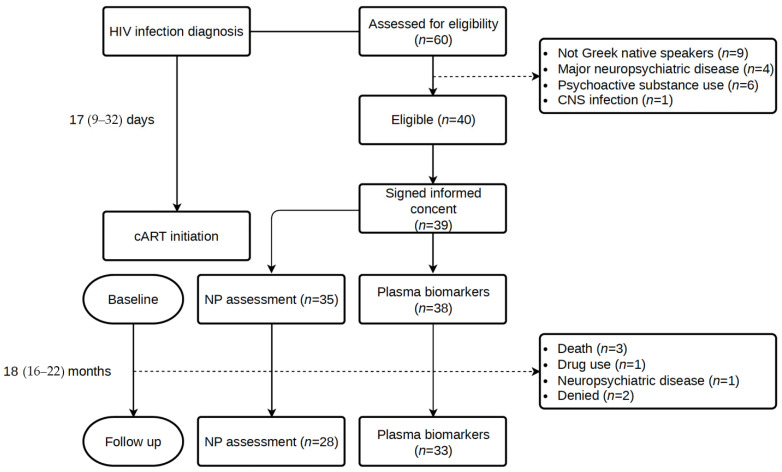
Study flow diagram.

A total of 60 consecutive participants newly diagnosed with HIV infection were assessed for eligibility, and 39 were included in the study. From HIV diagnosis to cART initiation, a median (IQR) of 17 (9–32) days elapsed, while the time until the initial assessment was 35 (12–63) days. The follow-up assessment took place at a median of 18 (IQR: 16–22) months after the initial evaluation. Figure 1 illustrates the study flow diagram with details about the number of individuals in each study visit and reasons for non-participation.

In total, 39 participants (37 males) were enrolled in the study, with a M = 37.7 ± SD = 10.7 years of age. Baseline demographics, HIV infection parameters, and comorbidities for the whole cohort, those who completed the 18-month follow-up neuropsychological assessment, and those who were lost to follow-up are listed in Table 2. Education level was high, with a median typical education of 14 years. Two participants were unable to undergo a comprehensive NP assessment in the baseline. Nine (23.1%) individuals presented with acute HIV infection (evidence of seroconversion < 6 weeks before diagnosis). Almost half of the participants were diagnosed at CDC stage 2 (CD4+ T cell count < 500 cells/μL and about 36% were late presenters with a CD4+ T cell count < 350 cells/μL, in accordance with Greek and European epidemiological data. Late presentation was more common among those lost to follow-up. The majority of the participants (82%) received an integrase inhibitor-based cART regimen, while the remaining 18% a protease-based regimen. No treatment changes were observed during the study period. Of note, during the early stages of the study, both regimens were considered first-line, according to the EACS guidelines, which have since been modified, with protease-containing regimens now being considered second-line treatment. Significant comorbidities, including cardiovascular disease, hypertension, and diabetes mellitus, were not present in our cohort of young PLWH, but smoking was prevalent in 67% of the participants. The baseline characteristics of those who underwent a follow-up assessment did not show statistically significant differences with those who were lost-to-follow-up. However, the latter were more commonly late presenters (55.5% vs. 28.6%) and had a lower median CD4+ T cell count (286 vs. 429 cells/μL), though these differences were not statistically significant considering the small sample size in each group. Three participants died in the period between baseline and follow-up (one due to complications resulting from Kaposi sarcoma, one due to cardiac arrest, and one due to unidentified causes).

### 3.2. Neuropsychological Assessment

**Figure 2:**
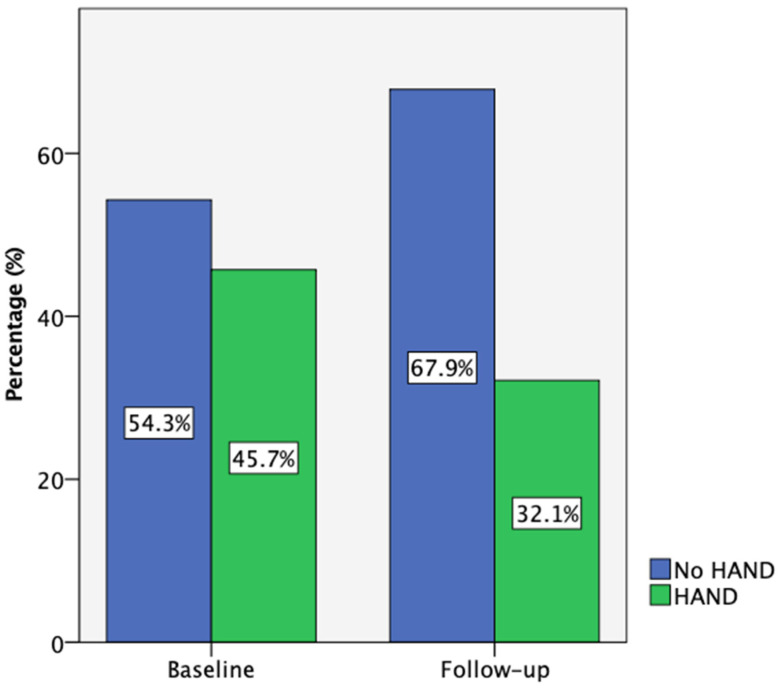
Participant HAND classification according to Frascati criteria at baseline (n = 35) and follow-up (n = 28).

At baseline, 35 participants completed the comprehensive neuropsychological assessment, while 28 (80%) returned for the 18-month follow-up. According to the Frascati criteria, 45.7% of participants met the criteria for HIV-associated neurocognitive disorders (HAND) at baseline, primarily with asymptomatic neurocognitive impairment (ANI). One individual presented with HIV-associated dementia (HAD) and was not able to complete this neurocognitive test battery. Also, one participant with low performance on cognitive tests reported neurocognitive symptoms at baseline and was categorized as having mild neurocognitive disorder (MND). Due to the small number of people in the MND and HAD categories, the data presented are referring to participants with and without HAND. By follow-up, this percentage decreased to 32.1% (Figure 2). Notably, three participants who initially had HAND improved to normal cognitive function, while none of those classified as neurocognitively normal at baseline progressed to HAND.

In the baseline assessment, 18 participants (51.4%) scored below 26 on the Montreal Cognitive Assessment (MoCA), indicating lower cognitive performance. By follow-up, this number decreased to 11 (39.3%). Significant improvements were observed in specific neuropsychological tests, including the Stroop Color and Word Test (+6.56 points, 95% CI: 1.3–11.8, *p* = 0.016, d = 0.52), phonemic fluency (+3.42 points, 95% CI: 0.29–6.6, *p* = 0.033, d = 0.44), and the Judgment of Line Orientation test (+1.15 points, 95% CI: 0.34–2.0, *p* = 0.007, d = 0.57). Importantly, no statistically significant cognitive decline was observed in any test over the 18-month period. Sensitivity analyses using LOCF yielded results consistent with those from complete-case analyses, suggesting that attrition did not substantially bias the neurocognitive outcome estimates (Table S1).

**Figure 3:**
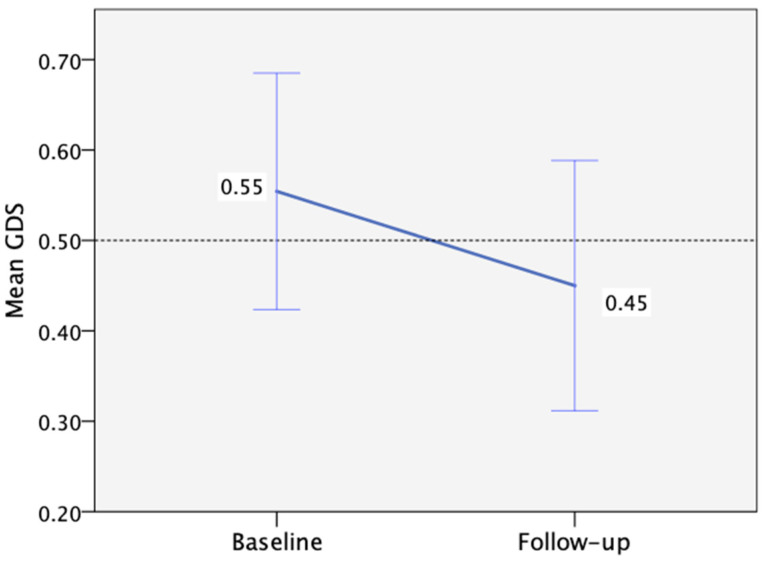
Mean Global Deficit Score change from baseline (n = 35) to follow-up (n = 28), with the threshold for low performance >0.50 (the error bars correspond to ±1 SE).

Regarding mental health at baseline, 6% and 26% of participants had high depression and anxiety scores (>7), respectively. However, no significant changes in depression or anxiety levels were observed between the baseline and follow-up visits (Table 3). Additionally, no correlation was identified between depression or anxiety levels and performance per cognitive domain. When considering overall cognitive performance as measured by the GDS, we observed a non-significant improvement from baseline to follow-up, with a mean change of −0.1 (95%CI: −0.27 to 0.05, *p* = 0.195, d = 0.25) (Figure 3).

**Figure 4:**
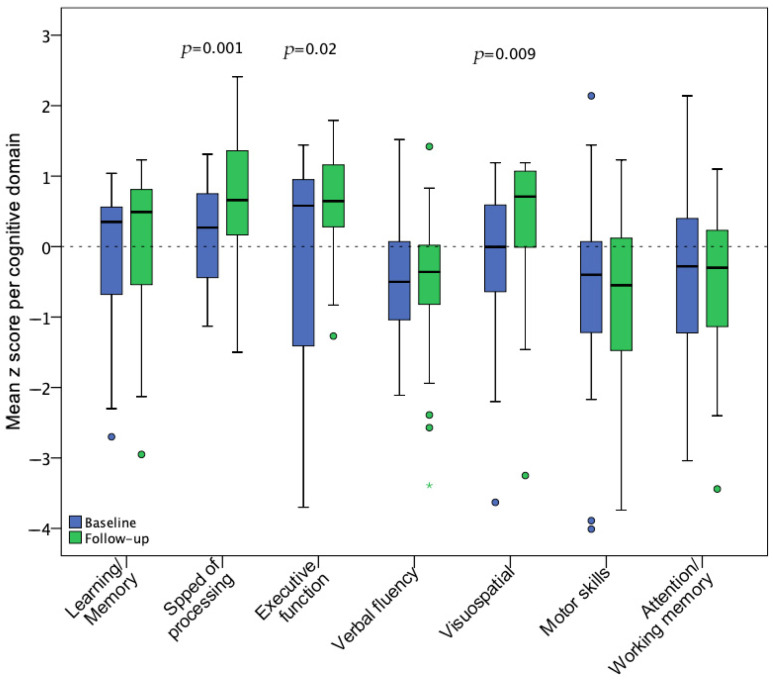
Mean z score changes for 7 cognitive domains from baseline (n = 35) to follow-up (n = 28) assessment. Statistical significance of changes was assessed with a paired *t*-test including the participants with available data for both visits (n = 28).

During the baseline assessment, the most affected cognitive domains were verbal fluency, fine motor dexterity, and attention/working memory, which did not show statistically significant improvement at the follow-up visit. In contrast, speed of information processing, executive function, and visuospatial ability significantly improved after 18 months. Figure 4 illustrates pre-post z score changes per cognitive domain.

### 3.3. Plasma Biomarkers

In all participants, plasma biomarkers associated with chronic inflammation and neurocognitive impairment were measured at baseline and at the 18-month follow-up. For four biomarkers (IL-1b, IL-6, IL-10, and S100B), the proportion of measurements below the level of detection (LOD) was >50%. In this case, further statistical analysis of changes over time was deemed not informative. The majority of the assessed biomarkers showed a statistically significant decrease at 18 months (TNFα, MCP1, sCD14, and sCD163), two remained unchanged (MIP-1b and NFL), while neopterin showed a statistically significant increase. Detailed levels and changes at the two time points are presented in Table 4.

### 3.4. Associations Between Plasma Biomarker Levels and Cognitive Performance

**Figure 5:**
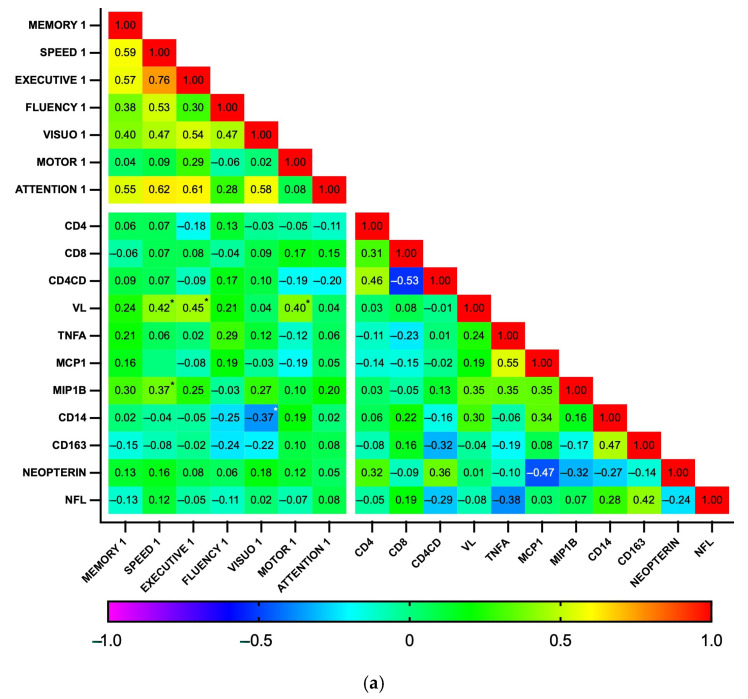

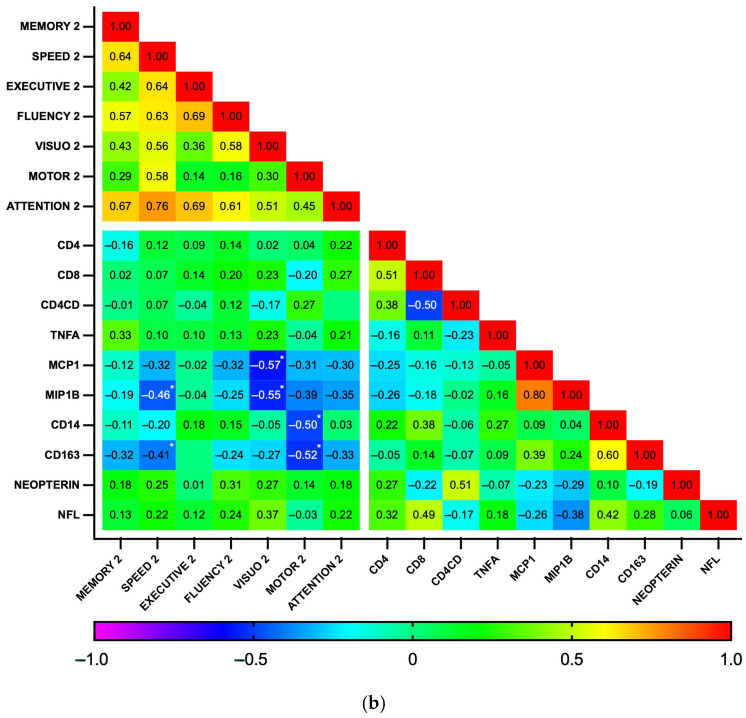
Correlation heatmap between plasma biomarkers and performance on cognitive domains at baseline (n = 35) (**a**) and follow-up (n = 28) (**b**). This heatmap diagrams the Spearman’s correlation coefficient *r* across the entire data set. For cognitive domains, the mean z score was calculated by averaging the scores of individual tests. Designated areas separated by spaces are intended to allow for better visualization of cognitive domains and plasma biomarkers. (*) Statistically significant correlations between biomarkers and cognitive domains.

Subsequently, correlations between biomarker levels and z-scores for each cognitive domain were explored using a heatmap (Figure 5). As expected, the analysis revealed a high degree of correlation among the seven cognitive domains, as well as between some biomarkers, particularly those related to general inflammation (IL-1b, IL-6, IL-10, and TNFα), at both time points. At baseline, no significant correlations were found between plasma biomarkers and cognitive domains. In contrast, at the 18-month follow-up, the biomarkers MCP1, MIP1b, CD14, and CD163 showed a pattern of negative correlation with cognitive function. Significant correlations were observed between MCP1 and MIP1b with visuospatial perception (*p* = 0.002 and 0.003, respectively) and between CD14 and CD163 with fine motor dexterity (*p* = 0.011 and 0.006, respectively). Additionally, MIP1b and CD163 had a statistically significant negative correlation with information processing speed (*p* = 0.014 and 0.031, respectively). Similarly, while GDS did not significantly correlate with any biomarker at baseline, GDS at 18 months positively correlated with MCP1b and CD163 (*p* = 0.018 for both), while individuals with GDS ≥0.5 had higher CD163 levels than those who were not impaired (364 vs. 274 ng/mL, *p* = 0.04, Cohen’s d = 0.90). Plasma biomarker levels did not otherwise differ between the HAND and the non-HAND groups, neither at baseline nor at follow-up (Table 5 and Table S2). PLWH with HAND at follow-up had significantly fewer years of education compared with PLWH without HAND.

## 4. Discussion

This is the first study to provide prospective data on neurocognitive health in a real-world cohort of newly diagnosed PLWH initiating cART in Greece. Neuropsychological performance and plasma biomarker trajectories were assessed over an 18-month period following cART initiation, showing improvements in select cognitive domains and in biomarkers of inflammation and macrophage activation. An exploratory analysis revealed a negative correlation between the cognitive performance and plasma biomarkers, but only at follow-up.

Frascati-defined HAND was present in 45.7% of participants at baseline, consistent with prior research, and was predominantly characterized by asymptomatic impairment. This finding was unexpected given the relatively young, otherwise healthy cohort of people with HIV, with minimal confounding or contributing factors for cognitive decline. Encouragingly, the prevalence of HAND decreased over time, accompanied by improvements in neurocognitive domains, particularly speed of information processing, executive function, and visuospatial ability. The Global Deficit Score, a quantitative measure of global cognition, showed a non-significant improvement following cART initiation. These findings suggest that early cART initiation may support neurocognitive recovery by mitigating the deleterious effects of HIV replication and immune activation in the CNS. However, the cognitive domains most affected, such as verbal fluency, fine motor dexterity, and attention/working memory did not show significant improvement.

Although significant improvements were observed in some cognitive measures at follow-up, the absence of a control group is a strong limitation for a causal inference regarding the possible effects of cART [27]. To assess the magnitude of these changes, within-subject effect sizes (Cohen’s d) were calculated, indicating a size range from small to moderate in tests showing significant improvement. These findings could suggest a combination of possible practice effects and possible cART-related benefits. It is important to highlight that the follow-up assessment was scheduled at 18 months after baseline, which constitutes an interval period that could reduce, but not eliminate, the influence of practice effects [28]. Nevertheless, even in the presence of practice effects, the indicated cognitive stability and/or improvement could be viewed positively. More specifically, in clinical populations at risk for neurocognitive decline, practice effects have been interpreted as indicators of preserved cognitive functioning. This interpretation is in line with evidence that individuals susceptible to practice effects tend to have better follow up cognitive performance over time than those who do not [29,30]. Consequently, while our findings should be interpreted cautiously due to the absence of a control group, the observed changes may still reflect clinically meaningful stability and/or improvement in cognitive functioning after cART initiation.

Few studies have assessed neurocognitive trajectories before and after cART initiation, particularly in the era of universally administering cART at diagnosis, independently of CD4+ T cell count. An earlier systematic review by Joska et al. included 15 prospective studies evaluating the effect of cART on cognitive function, with 11 showing improvement [31]. However, most of these studies focused on individuals with long-standing, advanced HIV infection, employed heterogeneous designs, and used varying cognitive assessments and methods of reporting of neurocognitive outcomes. Other prospective cohorts investigating HIV-associated NCI have predominantly included individuals already receiving cART [32,33,34] or did not specifically evaluate the impact of antiretroviral treatment on cognition [35]. The Strategic Timing of Antiretroviral Treatment (START) Neurology substudy, which compared cognitive trajectories in PLWH initiation cART immediately versus those deferring treatment, found no significant benefit of cART initiation in individuals with CD4+ T cell counts >500/μL [36]. Instead, both groups showed similar cognitive improvements over time, which the researchers attributed to practice effects resulting from the repeated administration of neuropsychological tests.

At baseline, plasma markers of inflammation and macrophage inflammation were elevated, consistent with the findings in people with untreated HIV infection [37]. Most biomarkers improved after 18 months of cART, but neopterin increased, and plasma NFL remained stable. Plasma NFL is a promising biomarker for diagnosing and monitoring HAND, as it correlates well with CSF levels and neurocognitive outcomes in PLWH. Anderson et al. suggested that plasma NFL levels decline with cART initiation; however, their study included only 16 participants not receiving cART at enrollment, with only 7 being treatment naïve [38]. In contrast, in our cohort, plasma NFL remained unchanged and was lower than levels reported in other treated PLWH cohorts [39,40,41]. Median baseline NFL concentrations were within the expected normal range, which could explain the absence of longitudinal change. A study that longitudinally assessed CSF NFL changes found a significant decrease after cART initiation in the total cohort but not in those who had normal CSF NFL levels at baseline [42]. Similarly, another study did not find a decrease in CSF NFL in those who had normal pretreatment levels, while affirming that increased CSF NFL levels are not common among neurocognitively asymptomatic PLWH with CD4+ counts above 200 cells/μL [40]. Stable plasma NFL levels in our cohort could suggest that there was no frank neurodegeneration or neuroaxonal injury among the study participants that is likely attributable to younger age, an absence of comorbidities, and a lower prevalence of advanced HIV infection (CD4+ T < 200 cells/μL) at diagnosis. However, the relatively short follow-up interval of 18 months might be insufficient to capture minimal but progressive axonal injury, especially in participants with subclinical impairment, for whom the sensitivity of plasma NFL may be limited. Moreover, while early treatment initiation likely mitigates axonal damage, we cannot definitively show that it prevents any NFL elevation without a matched control of people without HIV. However, the observed stability and low levels of plasma NFL is in line with the absence of clinical and cognitive deterioration over 18 months. Most HAND cases were classified as ANI, with only few cases presenting with more severe impairment, suggesting that neurocognitive dysfunction in this group may not be primarily driven by severe axonal damage. Notably, the only participant with HIV-associated dementia had an elevated plasma NFL level of 179 pg/mL, consistent with prior evidence linking NFL to severe neurodegeneration.

In contrast to markers of monocyte activation such as sCD14 and sCD163, which significantly decreased after cART initiation, plasma neopterin increased. This finding was unexpected, as previous studies have reported a significant decline in neopterin levels with cART [41,43,44]. This discrepancy prompted further investigation for the potential causes of neopterin increase in this cohort. Even though high assay specificity should be expected according to manufacturer data, cross-reactivity with structurally related pterins cannot be entirely excluded. Potential confounders, such as opportunistic infections and comorbidities, were not present among the participants in this study. In addition, we did not find any association of neopterin levels with any specific participant characteristic, such as age, acute HIV infection, late presentation, CD4+ count, and viral load. Of note, considering that the follow-up visit for most of the participants took place during the COVID-19 pandemic, SARS-CoV-2 vaccination or infection could have influenced neopterin levels [45]. What is more, neopterin, unlike other macrophage activation markers, reflects a distinct pathway of monocyte/macrophage activation, driven by interferon-gamma (IFN-γ). However, we did not find any other reason for persistent IFN-γ driven immune dysregulation specific to our cohort. Despite a large effect size (*r* = 0.54, *p* = 0.002) for the pre–post comparison, we cannot rule out that the observed neopterin increase represents a statistical artifact (Type I error). CSF neopterin levels have been shown to correlate with NCI severity in PLWH, and a direct pathophysiological link to neurodegeneration has been proposed through the release of cytokines and other inflammatory mediators [46]. However, neopterin levels in plasma may not accurately reflect CNS levels and could be linked to broader processes of persistent inflammation in PLWH.

Markers of monocyte activation showed a significant negative correlation with specific cognitive domains as well as global cognition, but only at the 18-month follow-up visit. The lack of correlation at baseline may be the result of confounding due to persistent HIV replication and systemic immune activation in untreated PLWH. Patterns of association become apparent as systemic inflammation diminishes with cART. This finding suggests the potential utility of plasma biomarkers in predicting cognitive impairment, but only in PLWH who have achieved virological suppression. In line with our findings, a previous study found a significant association between the monocyte activation markers CD14 and CD163 and worse cognitive performance in women living with HIV and viral suppression [47]. Moreover, PLWH with cognitive impairment (GDS ≥ 0.5) had higher plasma CD163 than those who were not impaired in a study of individuals with chronic HIV infection on cART [48]. Also consistent with our findings, a study of PLWH with detectable viral load did not detect any difference in plasma CD14 and CD163 levels between neurocognitively normal and impaired individuals [49].

This study has several strengths. It is the first prospective study to address a relatively unexplored HIV-related comorbidity in the Greek population of PLWH. Its prospective design enabled the assessment of within-person changes over time following the initiation of cART. We enrolled a cohort of otherwise healthy PLWH, free from significant comorbidities that could confound neurocognitive function. A detailed and comprehensive neuropsychological assessment was conducted, with strict adherence to the established Frascati criteria for defining cognitive deterioration, ensuring alignment with prior research. Additionally, we assessed a panel of plasma biomarkers and tracked their changes over an 18-month period. Our findings contribute to the limited literature on cognitive and biomarker changes after cART initiation, supporting the beneficial effects of early treatment and highlighting the potential role of plasma biomarkers in diagnosing HAND. Given that cognitive impairment in our cohort was predominantly asymptomatic, we were able to evaluate the often-debated category of ANI.

Our study is also subject to several limitations. First, this is a single-center study with a small number of participants, resulting in limited statistical power that increased vulnerability to Type II errors. This becomes even more important when considering the inherent heterogeneity in neurocognitive and biomarker measurements. The enrollment of only treatment-naïve PLWH and the strict inclusion and exclusion criteria did not allow for a larger sample. However, our cohort is homogeneous without comorbidities that could influence neurocognitive outcomes. To contextualize negative findings (*p*-value > 0.05) among cognitive and biomarker changes, we additionally incorporated, in our results, appropriate effect size measurements. In addition, the sample primarily included MSM, thus limiting the generalizability of the results in other populations of PLWH. In future studies, a multi-center design or an extended recruitment period is warranted to increase sample size, enhance generalizability, and improve the statistical power to detect clinically meaningful effects.

Second, we had a lost-to-follow-up rate of 20%, resulting from incident drug use, neuropsychiatric disease, or death, while only two participants refused to undergo a follow-up assessment. Although this attrition may introduce bias toward healthier individuals who remained engaged, the baseline characteristics of those lost to follow-up did not significantly differ from those of completers. However, there was a non-significant trend toward lower CD4+ counts and higher rates of late presentation in the lost-to-follow-up group, which may reflect limited statistical power to detect significant differences. This attrition could potentially lead to an underestimation of persisting cognitive impairment. Conversely, it is also plausible that individuals with more advanced disease or cognitive dysfunction could have a greater potential for improvement following cART initiation. A sensitivity analysis using the last observation carried forward imputation method yielded results consistent with our primary analysis for neurocognitive change.

Third, a strong limitation of our study is the use of unpublished normative data for several neuropsychological tests (BVMT, SDMT, JLO, LNS, and Spatial Span). Although these research norms were derived from a well-characterized control group matched to the clinical sample on key demographic variables, the lack of published and standardized normative references in Greece may influence the accuracy of calculated z-scores. As a result, the HAND classification might be affected, particularly in cases near diagnostic thresholds.

Fourth, the lack of CSF sampling is a major limitation when interpreting systemic biomarkers in relation to CNS outcomes. The search for sensitive and specific plasma biomarkers for the diagnosis and prognosis of neurodegenerative diseases is a rapidly advancing area of research. However, many candidate biomarkers may not accurately reflect intrathecal inflammation or CNS injury in situ, and their levels may be confounded by systemic inflammatory processes unrelated to CNS pathology. CSF biomarker analysis would add considerable depth in our analysis; however, we were mindful of not further increasing the assessment burden for study participants, which could have further reduced study participation rates.

Moreover, four key biomarkers (IL-1b, IL-6, IL-10, and S100B) were excluded from the analysis due to >50% values being below the assay LOD. The exclusion of these key inflammatory mediators represents a serious limitation that has affected the breadth of our results. Future studies should use ultrasensitive assays (e.g., Simoa, MSD) to capture low-level inflammation, especially when evaluating cART-treated populations.

Finally, while the established Frascati criteria were used to translate neuropsychological performance into a HAND diagnosis, this approach is not without limitations and has recently come under scrutiny for potentially overestimating HIV-associated NCI incidence [50].

## 5. Conclusions

In conclusion, this study provides valuable prospective data on neurocognitive health in a real-world cohort of newly diagnosed PLWH initiating cART in Greece. Our findings highlight improvements in select cognitive domains and biomarkers of immune activation following cART initiation. While HAND prevalence declined over time, certain cognitive deficits persisted, underscoring the complex pathways of neurocognitive recovery. The association between monocyte activation markers and cognitive performance, evident only after viral suppression, suggests a potential role for plasma biomarkers in predicting cognitive impairment. Despite its limitations, including the possible influence of practice effects on repeated neuropsychological testing, this study adds to the growing body of evidence on neurocognitive trajectories in PLWH and emphasizes the need for further research on the long-term impact of HIV and cART on brain health.

## Figures and Tables

**Table 1:** Neuropsychological test battery assessing general cognitive ability and seven cognitive domains.

	Test 1	Test 2
General Cognitive Ability	MoCA [14]	-
Memory and Learning	GVLT [15]	BVMT [16]
Speed of Information Processing	TMT-A [17]	SDMT [18]
Executive Functions	Stroop Test [19]	TMT-B [17]
Verbal Fluency	Semantic tasks [15]	Phonemic tasks [15]
Visuospatial Perception	JLO [20]	-
Motor Speed and Dexterity	Grooved Pegboard [21] (dominant hand)	Grooved Pegboard (non-dominant hand)
Attention and Working Memory	LNS [22]	Spatial Span [22] (forward and backward)

BVMT, Brief Visuospatial Memory Test; GVLT, Greek Verbal Learning Test; JLO, Judgement of Line Orientation; LNS, Letter Number Sequencing; MoCA, Montreal Cognitive Assessment; SDMT, Symbol Digit Modalities Test; TMT-A, Trail Making Test Part A; TMT-B, Trail Making Test Part B.

**Table 2:** Baseline cohort demographics, HIV infection characteristics, and comorbidities.

	All	Completed NP Follow-Up	Lost to Follow-Up	*p*-Value ^a^
**Baseline Demographics, HIV Infection Characteristics, and Comorbidities**	n = 39	n = 28	n = 9	
Age (years)	37.7 (±10.7)	39.1 (±13.6)	37.7 (±10.1)	0.776 ^^^
Male (%)	37 (94.9)	27 (96.4)	8 (88.9)	
White (%)	39 (100)	-	-	
Typical education (years)	14.5 (12.3–16)	12 (12–16)	14.5 (14–16)	0.30 ^#^
MSM (%) Heterosexual (%)	37 (94.9) 2 (5.1)	27 (96.4) 1 (3.6)	8 (88.9) 1 (11.1)	
CDC Classification, *n* (%)				0.10 ^§^
0	9 (23.1)	9 (32.1)	1 (11.1)	
1	5 (12.8)	3 (10.7)	3 (33.3)	
2	19 (48.7)	13 (46.4)	2 (22.2)	
3	6 (15.4)	3 (10.7)	3 (33.3)	
CD4 < 350 cells/μL, n (%)	14 (35.9)	8 (28.6)	5 (55.5)	0.229 ^§^
CD4+ count (cells/μL)	405 (260–506)	429 (302–513)	286 (139–500)	0.160 ^#^
CD8+ count (cells/μL)	1024 (620–1397)	1053 (621–1585)	942 (602–1293)	0.625 ^#^
CD4+/CD8+ ratio	0.34 (0.24–0.51)	0.36 (0.30–0.56)	0.32 (0.16–0.52)	0.286 ^#^
**HIV RNA (log_10_ copies/mL)**	5.0 (4.4–5.7)	5.0 (4.4–5.8)	4.6 (4.2–5.9)	0.648 ^#^
INSTI-based cART, n (%)	32 (82.1)			
PI-based cART, n (%)	7 (18.4)			
Smoking (ex or current), n (%)	26 (66.7)	19 (67.9)	7 (77.8)	0.695 ^§^

CDC, Centers of Disease Control and Prevention; HIV, human immunodeficiency virus; INSTI, integrase strand transfer inhibitor; MSM, men who have sex with men; NP, neuropsychological evaluation; PI, protease inhibitor; RNA, ribonucleic acid. ^a^ *p* values for comparison between those who completed and those who were lost to follow-up. ^#^ Mann–Whitney U test. ^§^ Fisher’s exact test. ^^^ Independent samples *t*-test.

**Table 3:** Participant performance per cognitive test at baseline and 18-month follow-up.

	All Participants	Participants with Follow-Up			
Test	Baseline (n = 35) (mean, SD)	Baseline (n = 28) (mean, SD)	Follow-Up (n = 28) (mean, SD)	*p* ^a^	Cohen’s d
**Screening**					
MoCA	25 (3.1)	25 (3.0)	24.9 (4.7)	0.84	−0.04
**Learning/Memory**					
GVLT Total Words	56.9 (9.7)	57.6 (10.0)	55.5 (11.4)	0.33	−0.19
BVMT-R Total Recall	24.1 (9.1)	25.2 (8.1)	25.9 (8.1)	0.70	0.08
GVLT Long Delay/Free Recall	13.3 (3.0)	13.6 (2.9)	13.5 (2.6)	0.81	−0.05
GVLT Long Delay/Cued Recall	13.6 (2.5)	13.8 (2.7)	13.8 (2.7)	0.90	0.02
BVMT Delayed Recall	10.1 (2.8)	10.1 (3.0)	9.8 (3.2)	0.21	−0.25
**Speed of information processing**					
TMT-A	32.2 (11.6)	31.5 (11.3)	28.0 (9.7)	0.07	−0.36
SDMT	49.8 (12.0)	50.3 (12.3)	53.8 (15.1)	0.10	0.32
**Executive function**					
TMT-B	84.1 (59.7)	78.6 (61.0)	59.5 (31.3)	0.06	−0.39
Stroop Color and Word	95.6 (20.2)	97.2 (19.1)	102.4 (16.0)	**0.02**	**0.52**
**Verbal fluency**					
Phonemic Fluency	32.6 (11.5)	31.6 (11.9)	35 (12.4)	**0.03**	**0.44**
Semantic Fluency	49.5 (9.1)	50.6 (9.8)	48.2 (10.1)	0.17	−0.28
**Visuospatial ability**					
Judgement of Line Orientation	16.6 (2.7)	16.8 (2.8)	18.0 (2.2)	**0.007**	**0.56**
**Motor dexterity**					
Grooved Pegboard (dominant hand)	72.4 (13.5)	72.0 (12.4)	69.2 (15.1)	0.25	−0.25
Grooved Pegboard (non-dominant hand)	79.5 (21.0)	81.6 (23.4)	77.8 (13.5)	0.34	−0.20
**Attention/Working memory**					
Spatial Span Forward	8.9 (2.6)	9.0 (2.8)	8.4 (1.9)	0.15	−0.28
Spatial Span Backward	7.4 (2.4)	7.4 (2.6)	7.3 (2.4)	0.76	−0.06
Letter-Number Sequencing	9.3 (2.8)	9.4 (3.0)	9.2 (2.9)	0.63	−0.09
**Mental health**					
HADS Depression, median (IQR) Score > 7, n (%) Anxiety, median (IQR) Score > 7, n (%)	2 (1–4.5) 2 (5.7) 4 (1–8) 9 (25.7)		1 (1–5) 4 (14.3) 4 (1–7) 5 (17.9)	0.67 ^#^ 0.43 ^#^	

BVMT, Brief Visuospatial Memory Test; GVLT, Greek Verbal Learning Test; HADS, Hospital Anxiety and Depression Scale; MoCA, Montreal Cognitive Assessment; TMT, Trail Making Test. ^a^ paired samples *t*-test, ^#^ Wilcoxon signed-rank test. Statistically significant comparisons appear in bold.

**Table 4:** Plasma biomarker levels at baseline and at follow-up.

Biomarker ^$^	LOD	<LOD (%) *	Baseline (n = 38)	Follow-Up (n = 33)	*p* ^#^	*r*
IL-1b	3.9	66.7	-	-	-	-
IL-6	9.4	63.9	-	-	-	-
IL-10	31.2	63.9	-	-	-	-
TNFa	15.6	25	12.6 (3.9–187.9)	3.9 (3.9–37.6)	**0.005**	**0.50**
MCP1	15.6	13.9	49.8 (28.5–76.1)	35 (28–47.5)	**0.006**	**0.47**
MIP-1b	15.6	16.7	26.7 (16.3–48.4)	33.4 (19.4–63.1)	0.851	0.03
sCD14	62.5	0	1620 (1196–2129)	858 (553–1154)	**<0.001**	**0.68**
sCD163	156	0	627 (506–830)	288 (239–374)	**<0.001**	**0.86**
S100B	46.9	63.9	-	-	-	-
Neopterin	0.156	0	10.6 (7–13.9)	13 (9.9–17)	**0.002**	**0.54**
NFL	1.59	0	7.5 (5.5–10.6)	7.2 (5.7–9.1)	0.524	0.11

IL, interleukin; TNFa, tumor necrosis factor alpha; MCP1, monocyte chemoattractant protein-1; MIP-1b, macrophage inflammatory protein-1beta; sCD, soluble cluster of differentiation; NFL, neurofilament light chain; LOD, level of detection. ^$^ All values are reported in pg/mL, except for sCD14, sCD163, and neopterin which are reported in ng/mL. ^#^ Wilcoxon signed-rank test *p*-value and effect size (*r*). * When >50% biomarker measurements were below the LOD, further analysis was not pursued. Statistically significant comparisons appear in bold.

**Table 5:** Participant characteristics and plasma biomarkers between neurocognitively normal and impaired individuals, at baseline and at 18 months.

	Baseline				Follow-Up			
	Non-HAND	HAND	*d/r* ^^^	*p*	Non-HAND	HAND	*d/r*	*p*
Age (y) ^$^	34.3 (10.4)	41.2 (10.2)	0.67	0.06	35.2 (9.9)	42.9 (8.9)	0.8	0.06
Education (y)	14.8 (1.9)	13.7 (3.4)	0.43	0.24	15.6 (1.8)	12.2 (3.3)	**1.42**	**0.002**
CD4+ (cells/μL)	432 (196)	379 (170)	0.28	0.41	809 (248)	807 (458)	0.01	0.99
CD8+ (cells/μL)	1296 (958)	951 (460)	0.46	0.20	1041 (451)	959 (542)	0.17	0.68
CD4+/CD8+ ratio	0.99 (0.4)	0.85 (0.5)	0.08	0.37	0.86 (0.35)	0.90 (0.42)	0.09	0.82
HIV RNA (log_10_ copies/mL)	5.15 (0.83)	4.96 (1.3)	0.19	0.61	-	-		-
TNFa ^#^	21.5 (6–189)	11.1 (3–92)	0.17	0.31	18 (3.9–158)	3.9 (3.9–8)	0.30	0.11
MCP1	49.8 (28–76)	52.1 (30–76)	0	1.0	42 (31–49)	27.6 (19–37)	0.30	0.11
MIP-1b	35.5 (17–77)	23.1 (12–31)	0.25	0.15	38 (22–65)	34 (19–102)	0.02	0.88
sCD14	1534 (1124–2091)	1646 (1205–2590)	0.13	0.45	787 (511–1167)	858 (514–1133)	0.05	0.79
sCD163	626 (505–860)	630 (519–821)	0.06	0.72	277 (208–320)	295 (262–402)	0.17	0.36
Neopterin	11.2 (7–14)	10 (7–14)	0.01	0.97	13.3 (10–20)	13 (10–17)	0.08	0.68
NFL	7.6 (6–11)	7.2 (6–11)	0.03	0.85	7.7 (6–10)	6.6 (5–9)	0.23	0.23

HAND, HIV-associated neurocognitive disorder; MCP1, monocyte chemoattractant protein-1; MIP-1b, macrophage inflammatory protein-1beta; NFL, neurofilament light chain; sCD, soluble cluster of differentiation; TNFa, tumor necrosis factor alpha. ^^^ Cohen’s *d* was used to calculate the effect sizes for parametric and *r* for non-parametric statistical testing. ^$^ Age, education, CD4+, CD8+, CD4+/CD8+ ratio, and HIV-RNA are expressed as mean±SD, and the independent *t*-test was used for between-groups comparisons. Statistically significant comparisons appear in bols. ^#^ Plasma biomarkers are expressed in median (IQR) and the Mann–Whitney U test was used for between-groups comparisons. Values are reported in pg/mL, except for sCD14, sCD163, and neopterin which are reported in ng/mL.

## Data Availability

The raw data supporting the conclusions of this article will be made available by the authors on request.

## Supplementary Materials

The following supporting information can be downloaded at: https://www.mdpi.com/article/10.3390/biomedicines13071704/s1, Table S1. Participant performance per cognitive test and cognitive domain at baseline and 18-month follow-up using the last observation carried forward imputation method; Table S2. Baseline and follow-up plasma biomarkers changes for neurocognitively normal and impaired individuals.
