# Supplementary material for: Prospective Neuropsychological and Plasma Biomarker Changes in Treatment-Naïve People Living with HIV After Antiretroviral Treatment Initiation
Source: Biomedicines. 2025 Jul 12;13(7):1704. doi: 10.3390/biomedicines13071704 (PMC12292645; doi:10.3390/biomedicines13071704)
Supplement: Supplementary file 1 [file biomedicines-13-01704-s001.zip › biomedicines-3674577-supplementary.pdf]

Table S1. Participant performance per cognitive test and cognitive domain at baseline and 18-month follow-up using the last observation carried forward imputation method.

Test	Baseline (n=35) (mean, SD)	Follow-up (n=35) (mean, SD)	*p*[a]	Cohen's *d*
MoCA	25 (3.1)	24.9 (4.7)	0.84	-0.04
GVLT Total Words	56.9 (9.7)	55.2 (10.7)	0.33	-0.17
BVMT-R Total Recall	24.1 (9.1)	25.0 (8.4)	0.69	0.07
GVLT Long Delay / Free Recall	13.3 (3.0)	13.2 (2.7)	0.81	-0.04
GVLT Long Delay / Cued Recall	13.6 (2.5)	13.5 (2.7)	0.90	-0.02
BVMT Delayed Recall	10.1 (2.8)	9.8 (3.0)	0.21	-0.22
TMT-A[#]	32.2 (11.6)	29.3 (10.7)	0.07	-0.32
SDMT	49.8 (12.0)	52.6 (14.5)	0.10	0.29
TMT-B[#]	84.1 (59.7)	67.3 (40.5)	0.06	-0.34
Stroop Color and Word	95.6 (20.2)	101.4 (17.2)	**0.02**	**0.46**
Phonemic Fluency	32.6 (11.5)	35.3 (11.6)	**0.03**	**0.39**
Semantic Fluency	49.5 (9.1)	48.1 (9.2)	0.17	-0.25
Judgement of Line Orientation	16.6 (2.7)	17.6 (2.3)	**0.008**	**0.48**
Grooved Pegboard[#] (dominant hand)	72.4 (13.5)	69.9 (15.7)	0.24	-0.22
Grooved Pegboard[#] (non-dominant hand)	79.5 (21.0)	77.7 (13.7)	0.34	-0.18
Spatial Span Forward	8.9 (2.6)	8.4 (1.9)	0.15	-0.26
Spatial Span Backward	7.4 (2.4)	7.3 (2.2)	0.76	-0.05
Letter-Number Sequencing	9.3 (2.8)	9.2 (2.6)	0.63	-0.08
Memory	0 (1.1)	0.1 (1.1)	0.42	0.14
Speed of processing	0.19 (0.72)	0.51 (0.87)	**0.001**	**0.60**
Executive function	-0.30 (1.68)	0.13 (1.42)	**0.04**	**0.37**
Fluency	-0.52 (0.85)	-0.48 (1.0)	0.74	0.06
Visuospatial ability	-0.24 (1.0.7)	0.10 (1.07)	**0.01**	**0.47**
Motor dexterity	-0.74 (1.25)	-0.76 (1.36)	0.84	-0.04
Attention/ Working memory	-0.40 (1.19)	-0.56 (1.03)	0.25	-0.21

BVMT, Brief visuospatial memory test; GVLT, Greek verbal learning test; HADS, Hospital anxiety and depression scale; MoCA, Montreal cognitive assessment; TMT, trail making test.

[α] paired samples t-test

[#] For TMT-A, TMT-B, and Grooved Pegboard higher raw scores mean worse performance.

Table S2. Baseline and follow-up plasma biomarkers changes for neurocognitively normal and impaired individuals.

	Non-HAND (n=18)			HAND (n=16)		
	Baseline	Follow-up	p	Baseline	Follow-up	p
CD4+ (cells/μL)	447 (188)	824 (233)	**<0.001**	379 (170)	739 (397)	**<0.001**
CD8+ (cells/μL)	1321 (981)	970 (456)	0.129	951 (460)	953 (490)	0.99
CD4+/CD8+ ratio	0.50 (0.37)	0.98 (0.42)	**<0.001**	0.52 (0.55)	0.85 (0.46)	**0.02**
TNFa[#]	21.5 (6-189)	9.7 (3.9-183)	0.07	11.1 (3-92)	3.9 (3.9-19)	**0.04**
MCP1	49.8 (28-76)	42 (33-56)	**0.04**	52.1 (30-76)	31.8 (27-341)	0.06
MIP-1b	35.5 (17-77)	34.4 (19-64)	0.381	23.1 (12-31)	33.3 (19-61)	0.30
sCD14	1534 (1124-2091)	787 (563-1192)	**0.03**	1646 (1205-2590)	863 (473-1160)	**0.001**
sCD163	626 (505-860)	277 (200-361)	**<0.001**	630 (519-821)	292 (265-375)	**<0.001**
Neopterin	11.2 (7-14)	13.3 (10-23)	**0.004**	10 (7-14)	12.7 (10-17)	0.20
NFL	7.6 (6-11)	8.3 (6-10)	0.438	7.2 (6-11)	6.3 (5-8)	0.14

HAND, HIV-associated neurocognitive disorder; MCP1, monocyte chemoattractant protein-1; MIP-1b, Macrophage inflammatory protein-1beta; NFL, neurofilament light chain; sCD, soluble cluster of differentiation; TNFa, tumor necrosis factor alpha.

[$] CD4+, CD8+, and CD4+/CD8+ ratio are expressed as mean±SD, and the paired t-test was used for pre-post comparisons.

[#] Plasma biomarkers are expressed in median (IQR) and the Wilcoxon signed-rank test was used for pre-post comparisons. Values are reported in pg/ml, except for sCD14, sCD163, and neopterin which are reported in ng/ml.
